# Reactive Inorganic Vapor Deposition of Perovskite Oxynitride Films for Solar Energy Conversion

**DOI:** 10.34133/2019/9282674

**Published:** 2019-11-11

**Authors:** Tao Fang, Huiting Huang, Jianyong Feng, Yingfei Hu, Qinfeng Qian, Shicheng Yan, Zhentao Yu, Zhaosheng Li, Zhigang Zou

**Affiliations:** ^1^Collaborative Innovation Center of Advanced Microstructures, National Laboratory of Solid State Microstructures, Department of Physics, Nanjing University, 22 Hankou Road, Nanjing 210093, China; ^2^College of Engineering and Applied Sciences, Nanjing University, 22 Hankou Road, Nanjing 210093, China; ^3^Jiangsu Key Laboratory for Nano Technology, Nanjing University, 22 Hankou Road, Nanjing 210093, China

## Abstract

The synthesis of perovskite oxynitrides, which are promising photoanode candidates for solar energy conversion, is normally accomplished by high-temperature ammonolysis of oxides and carbonate precursors, thus making the deposition of their planar films onto conductive substrates challenging. Here, we proposed a facile strategy to prepare a series of perovskite oxynitride films. Taking SrTaO_2_N as a prototype, we prepared SrTaO_2_N films on Ta foils under NH_3_ flow by utilizing the vaporized SrCl_2_/SrCO_3_ eutectic salt. The SrTaO_2_N films exhibit solar water-splitting photocurrents of 3.0 mA cm^−2^ at 1.23 V vs. RHE (reversible hydrogen electrode), which increases by 270% compared to the highest photocurrent (1.1 mA cm^−2^ at 1.23 V vs. RHE) of SrTaO_2_N reported in the literature. This strategy may also be applied to directly prepare a series of perovskite oxynitride films on conductive substrates such as ATaO_2_N (A = Ca, Ba) and ANbO_2_N (A = Sr, Ba).

## 1. Introduction

Perovskite oxynitrides, which may have the advantages of both oxides and nitrides [1], emerge as an important class of materials due to their promising applications in pigments [2], dielectrics [3, 4], ferroelectrics [5], colossal magneto-resistance [6], and photocatalysis [7, 8]. They usually have smaller bandgap than those of prototypical oxides and better chemical stability than those of prototypical nitrides. Perovskite oxynitrides are currently predicted to be promising candidates for photoelectrochemical (PEC) water splitting [9], which is a potential way to harness solar energy [10–19].

To date, only some particle-assembled oxynitride films (such as LaTiO_2_N, BaTaO_2_N, SrTaO_2_N, and LaTaON_2_) have been investigated for PEC water splitting [20–24]. In order to prepare the particle-assembled oxynitride films, the oxynitride powders are firstly synthesized by nitridation of corresponding oxide precursors at high temperature under ammonia flow usually at 800~1300°C [5, 25–29]. Subsequently, the particle-assembled oxynitride films are prepared by electrophoretic deposition or particle transfer methods [20–24]. However, these particle-assembled films often suffer from poor charge carrier transport not only among film particles but also between the film particles and the conductive substrate. Also, the efficiency loss may even occur at the exposed region of the underlying conductive substrate [30, 31]. Various efforts have been made to improve their PEC performances, such as increasing the particle crystallinity or H_2_ annealing to prompt the charge transport in film particles, and using necking treatment to ameliorate the charge transport among film particles [20–24]. Unfortunately, the electron-hole recombination at the interface between the film particles and conductive substrate is still serious in these particle-assembled films, thus hampering their solar-to-hydrogen efficiency.

It is indispensable and challenging to directly synthesize oxynitride photoanode films on a conductive substrate for overcoming the main shortcomings of the particle-assembled films, including the poor charge carrier transport and the efficiency loss at the exposed conductive substrate. Here, we have presented a strategy of reactive inorganic vapor deposition and prepared a series of oxynitride films, for example, SrTaO_2_N. The as-prepared SrTaO_2_N photoanode films exhibit a significant increase in the photocurrent as well as photochemical stability and a cathodic shift in onset potential for water oxidation, in comparison with the particle-assembled photoanode films.

## 2. Results

**Figure 1:**
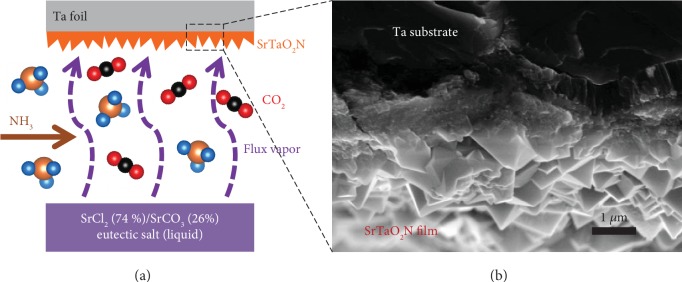
Schematic illustration. (a) Schematic illustration of the reactive inorganic vapor deposition method. (b) Cross-sectional SEM image of the as-prepared SrTaO_2_N film.

In this study, we have prepared SrTaO_2_N photoanode films by reactive inorganic vapor deposition, as illustrated in Figure 1(a). SrCl_2_ (74%)/SrCO_3_ (26%) eutectic salt is used to generate SrCl_2_ vapor during the nitridation process and provide an Sr source for the formation of SrTaO_2_N. The CO_2_ released from SrCO_3_ upon the nitridation process is employed as an oxidant to oxidize Ta to Ta^5+^ [32]. Ta foil is used both as a Ta source and as a conductive substrate. The SrCl_2_/SrCO_3_ eutectic salt may be evaporated during the ammonolysis process at 950°C for 2 hours, which is approximately 280°C higher than their melting temperature, according to the phase diagram in [Supplementary-material supplementary-material-1] [33]. The vaporized flux and released CO_2_ react with the Ta foils under the ammonia atmosphere, resulting in the formation of SrTaO_2_N film on Ta foil. The morphology of the SrTaO_2_N films was analyzed using field-emission scanning electron microscopy (SEM). The top-down image ([Supplementary-material supplementary-material-1]) and cross-sectional image (Figure 1(b)) show that the SrTaO_2_N film is a crack-free film consisting of compact and octahedron-like grains; the close contact between the SrTaO_2_N film and Ta substrate is propitious to charge carrier transport.

**Figure 2:**
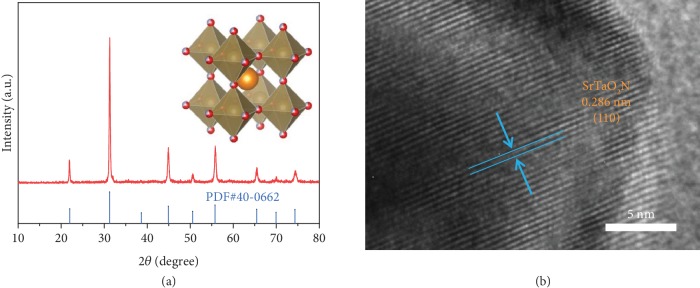
XRD pattern and HRTEM image. XRD pattern (a) and HRTEM image (b) of the as-prepared SrTaO_2_N film. The inset shows the crystal structure of SrTaO_2_N.

The X-ray diffraction (XRD) pattern (shown in Figure 2(a)) confirms the synthesis of the SrTaO_2_N film, based on the JCPDS file (PDF#40-0662). The high-resolution transmission electron microscopy (HRTEM) image of Figure 2(b) displays a lattice spacing of 0.286 nm, corresponding to the SrTaO_2_N (1 1 0) facet. The HRTEM image and selected area electron diffraction (SAED) pattern ([Supplementary-material supplementary-material-1]) suggest that the SrTaO_2_N samples exhibit high crystallinity, which favors electron transport.

**Figure 3:**
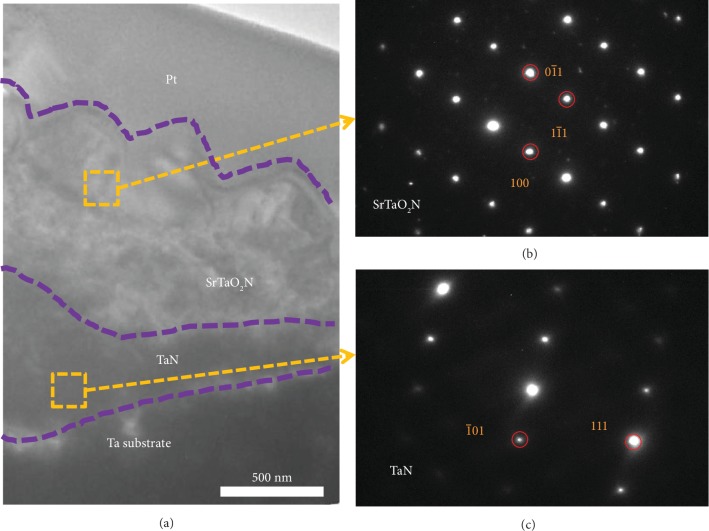
Cross-sectional TEM and SAED images of the SrTaO_2_N film. Cross-sectional TEM image (a) and SAED images (b, c) at different depths of the SrTaO_2_N film.

The cross section of the SrTaO_2_N film was further studied by TEM. As shown in Figure 3(a), the formation of a TaN subnitride layer can be observed between the SrTaO_2_N film and Ta substrate, which is suggested by selected area electron diffraction (SAED) images (Figures 3(b) and 3(c)). The presence of a highly conductive TaN phase provides an efficient electron transfer pathway that benefits the electron transfer from the SrTaO_2_N film to Ta substrate. The electrical conductivity of as-prepared SrTaO_2_N film photoanode is estimated to be 1.82 × 10^−5^ S cm^−1^ (derived from [Supplementary-material supplementary-material-1]), which is approximately 23.6 times of 0.77 × 10^−6^ S cm^−1^ for the particle-assembled SrTaO_2_N photoanode [34]. The SrTaO_2_N films prepared by our method exhibit much higher conductivity than the particle-assembled films, which may be ascribed to the reduction in the interfacial resistance.

**Figure 4:**
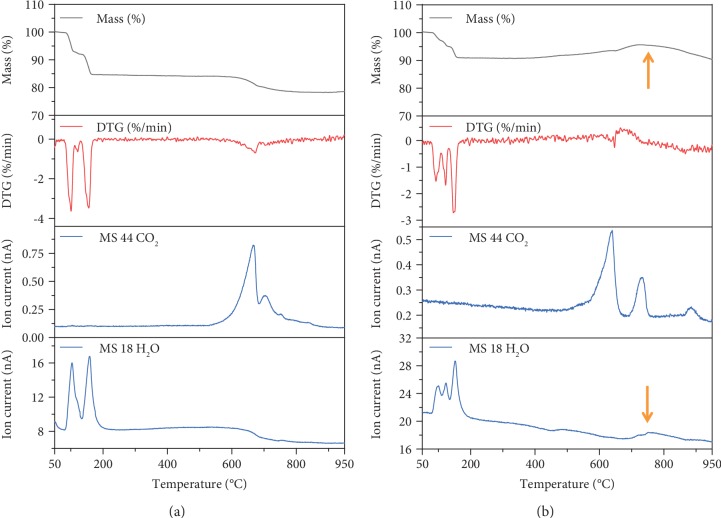
The combination of TG-DTA and mass spectroscopy. Thermogravimetric analysis (TG), differential thermal gravity (DTG), and mass spectra of the SrCl_2_/SrCO_3_ eutectic salt (26 mol% SrCO_3_, 74 mol% SrCl_2_·6H_2_O) (a) and the SrCO_3_/SrCl_2_ eutectic salt+Ta (the mass ratio of the eutectic salt and the Ta powder is 2 : 1) (b).

The combination of TG-DTA (thermogravimetric and differential thermal analysis) and mass spectral measurements for the SrCO_3_/SrCl_2_ eutectic salt sample and SrCO_3_/SrCl_2_ eutectic salt+Ta sample was carried out to study the formation mechanism of SrTaO_2_N, as shown in Figure 4. The weight loss in the temperature range of approximately 70°C to 170°C is caused by the loss of crystal water in SrCl_2_/SrCO_3_ eutectic salt, which is confirmed by the H_2_O signal in mass spectra. SrCO_3_ started to decompose in the temperature range of approximately 550°C to 800°C, according to the weight loss (Figure 4(a)) and CO_2_ signal in mass spectra. Compared with the SrCl_2_/SrCO_3_ eutectic salt sample, the SrCO_3_/SrCl_2_ eutectic salt+Ta sample exhibited a weight increase and a peak of H_2_O signal in mass spectra around 750°C, which may be an evidence of nitridation reaction.

Control experiments were also carried out by nitriding Ta foils in the same condition with different components of the flux reagent. In the case of the SrCl_2_ flux reagent, only small amount of TaN_0.04_ and Ta_2_N was obtained, as shown in the XRD pattern of [Supplementary-material supplementary-material-1]. Without the presence of carbonate, there is no CO_2_ released during the nitridation process, metallic Ta cannot be nitrided to high-oxidation-state nitride directly by ammonia upon this condition, which is consistent with literatures [32, 35]. Using only SrCO_3_ as the flux reagent, Ta_3_N_5_ instead of SrTaO_2_N was obtained ([Supplementary-material supplementary-material-1]). These results indicate that both the SrCl_2_ flux vapor and CO_2_ released from SrCO_3_ are crucial for the formation of SrTaO_2_N.

There are no chlorine impurities left on the SrTaO_2_N film, according to XPS spectra ([Supplementary-material supplementary-material-1]). The carbon in the films was analyzed by XPS, as shown in [Supplementary-material supplementary-material-1]. The C1s spectra of the SrTaO_2_N film show a peak at 284.6 eV, corresponding to the surface adventitious carbon. And the TEM and HRTEM images ([Supplementary-material supplementary-material-1]) suggested that there is no amorphous carbon on the surface of SrTaO_2_N particles. Therefore, the reaction equation for the formation of SrTaO_2_N films may be written as follows:

(1) $$\begin{array}{c} 4\text{Ta}+4{\text{SrCl}}_{2}+5{\text{CO}}_{2}+4{\text{NH}}_{3}⟶4{\text{SrTaO}}_{2}\text{N}+8\text{HCl}+5\text{C}+2{\text{H}}_{2}\text{O} \end{array}$$

In order to find the optimal composition of the SrCl_2_/SrCO_3_ flux reagent for the synthesis of SrTaO_2_N film photoanodes, control experiments were also carried out by nitriding Ta foils in the same condition with different compositions of the SrCl_2_/SrCO_3_ flux reagent (50%/50%, 74%/26%, and 80%/20%). The SrTaO_2_N film photoanode prepared with SrCl_2_ (74%)/SrCO_3_ (26%) eutectic salt exhibits the best PEC performance, as shown in [Supplementary-material supplementary-material-1]. The photocurrent of SrTaO_2_N photoanode prepared with the SrCl_2_ (50%)/SrCO_3_ (50%) flux reagent at 1.23 V vs. RHE (2.0 mA cm^−2^) is only 66.7% of that of SrTaO_2_N photoanode prepared with SrCl_2_ (74%)/SrCO_3_ (26%) eutectic salt (3.0 mA cm^−2^). The melting temperatures of the SrCl_2_ (50%)/SrCO_3_ (50%) flux reagent, SrCl_2_ (74%)/SrCO_3_ (26%) eutectic salt and SrCl_2_ (80%)/SrCO_3_ (20%) flux reagent are 870°C, 670°C and 715°C, respectively, according to the phase diagram in [Supplementary-material supplementary-material-1]. When using the SrCl_2_ (50%)/SrCO_3_ (50%) flux reagent as a precursor, approximately half of the flux reagent remained in the alumina combustion boat after the 2 hours of the nitridation process. These results suggested that the SrCl_2_ (74%)/SrCO_3_ (26%) eutectic salt with the lowest melting temperature may introduce the highest Sr partial pressure during the nitridation process, thus resulting in the best PEC performance of SrTaO_2_N film photoanode.

**Figure 5:**
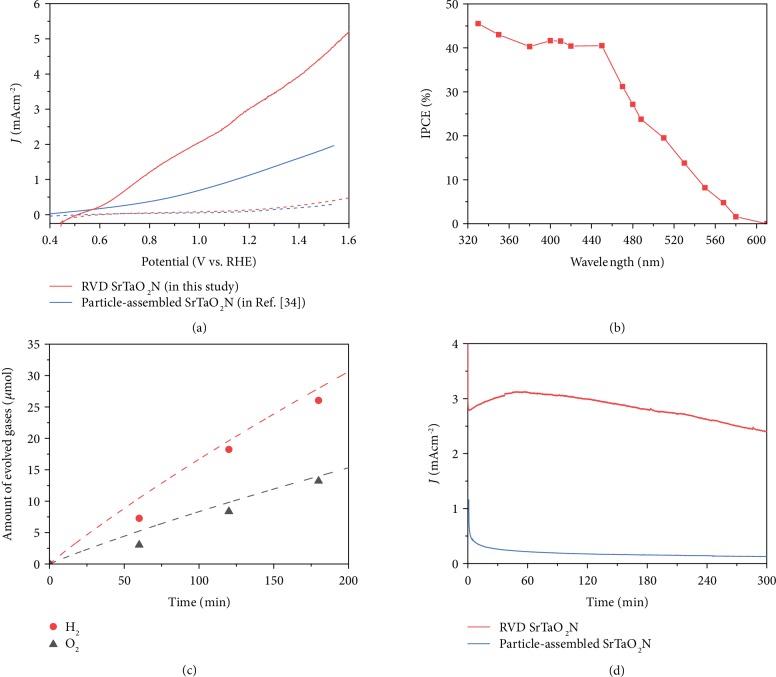
PEC performance and faradaic efficiency. (a) Photocurrents of the directly prepared SrTaO_2_N film and particle-assembled SrTaO_2_N photoanodes [34] in 1 M NaOH (pH = 13.6) electrolyte under AM 1.5G (100 mW cm^−2^) simulated sunlight and a scan rate of 30 mV s^−1^. (b) Wavelength dependence on the incident photon-to-current conversion efficiency (IPCE) of the SrTaO_2_N film photoanode with the Co/CoOOH catalyst layer measured at 1.23 V vs. RHE in 1 M NaOH (pH = 13.6) aqueous solution. (c) Gas chromatography for O_2_ and H_2_ generated from the SrTaO_2_N film photoanode modified with the Co/CoOOH layer and Pt counter electrode during PEC water splitting at an applied potential of 1.23 V vs. RHE for 180 min. (d) *I*-*t* curve for the SrTaO_2_N film photoanode and particle-assembled SrTaO_2_N photoanodes during PEC water splitting at 1.23 V vs. RHE (for 240 min) under AM 1.5G (100 mW cm^−2^) illumination.

Co/CoOOH catalyst layers were deposited onto the SrTaO_2_N photoanode films by a two-step electrodeposition method [36]. A photoassisted electrodeposition method [37] was adopted to produce a conformal CoOOH layer on the SrTaO_2_N film by deposition on only where photoinduced holes were generated. The SEM image indicates the conformal distribution of the CoOOH layer on the SrTaO_2_N photoanodes ([Supplementary-material supplementary-material-1]). As illustrated in Figure 5(a), with the surface decoration Co/CoOOH catalyst layers, the SrTaO_2_N film photoanode synthesized by the reactive inorganic vapor deposition method (RVD SrTaO_2_N) exhibits a solar photocurrent of 3.0 mA cm^−2^ at 1.23 V vs. RHE and a low onset potential for water oxidation at 0.55 V vs. RHE. The highest PEC performance of particle-assembled SrTaO_2_N photoanodes (particle-assembled SrTaO_2_N) in the literature exhibits a solar photocurrent of 1.1 mA cm^−2^ at 1.23 V vs. RHE and an onset potential of 0.6 V vs. RHE for water oxidation (Figure 5(a), blue curves) [34]. Compared with the particle-assembled SrTaO_2_N photoanode, the directly prepared SrTaO_2_N film photoanode shows a ca. 270% enhancement in the photocurrent at 1.23 V vs. RHE and a ca. 50 mV cathodic shift in onset potential for water oxidation. The wavelength dependence of the incident photon-to-current conversion efficiency (IPCE) of the SrTaO_2_N film photoanode is shown in Figure 5(b). The onset wavelength for the photocurrents is about 600 nm, which agrees well with the optical absorption edge of SrTaO_2_N ([Supplementary-material supplementary-material-1]). The integrated photocurrent is 2.9 mA cm^−2^ at 1.23 V vs. RHE, which is calculated by multiplication of the measured IPCE spectra with the AM 1.5G standard solar spectral distribution ([Supplementary-material supplementary-material-1]). It agrees well with the experimental photocurrent of 3.0 mA cm^−2^ at 1.23 V vs. RHE in Figure 5(a). The small difference between the integrated and experimental photocurrents suggests that the IPCE spectra and the photocurrent under AM 1.5G illumination are credible.

The applied bias photon-to-current efficiency (ABPE) of the SrTaO_2_N films calculated by using the *J*-*V* curve (Figure 5(a)) is shown in [Supplementary-material supplementary-material-1], assuming 100% faradaic efficiency. The maximum ABPE is 0.53% at 0.85 V vs. RHE for SrTaO_2_N film photoanode, which is ca. 3.5 times higher than that of 0.15% at 0.9 V vs. RHE for particle-assembled SrTaO_2_N photoanode. Gas chromatography was used to demonstrate that H_2_ and O_2_ gases evolved during PEC water splitting in a stoichiometric ratio of 2 : 1, and the faradaic efficiency was 93% for H_2_ and 94% for O_2_, respectively (Figure 5(c)). The band gap and flat band potentials (*E*_fb_) of the SrTaO_2_N film photoanode were investigated by the Tauc plot ([Supplementary-material supplementary-material-1]) and Mott-Schottky plots ([Supplementary-material supplementary-material-1]), respectively. These results show that the band gap of SrTaO_2_N is approximately 2.1 eV, and the conduction and valence band edges for SrTaO_2_N are estimated to be -0.5 and 1.6 V vs. RHE, respectively, which agrees well with previous report [38].

Photochemical stability is a vital parameter for the application of solar water splitting. The SrTaO_2_N film photoanode exhibits an initial photocurrent of approximately 2.8 mA cm^−2^ and retains approximately 86% of its initial activity after AM 1.5G (100 mW cm^−2^) irradiation for 5 hours, which shows significant improvement compared with the particle-assembled SrTaO_2_N photoanodes, as shown in Figure 5(d). The improvement in the stability may be ascribed to not only the close contact between SrTaO_2_N grains but also the highly conductive TaN phase benefiting the electron transfer from the SrTaO_2_N film to Ta substrate. The gradual drop of the photocurrent is partly caused by the peeling off and the dissolution of the CoOOH layer in strong base (1 M NaOH, pH = 13.6) during the stability testing ([Supplementary-material supplementary-material-1]). Similar phenomenon has also been observed that the CoOOH electrocatalyst shows a steady decrease in the current density for oxygen evolution reaction in 1 M KOH [36].

**Figure 6:**
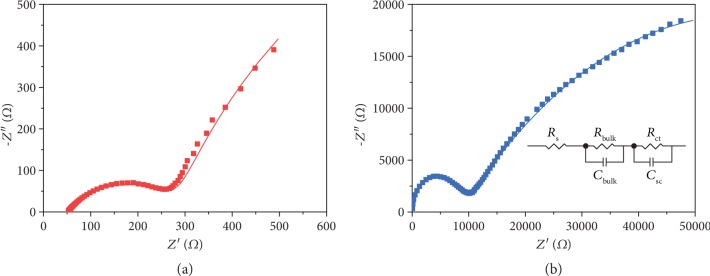
Electrochemical impedance spectroscopy measurements. Nyquist plots of the SrTaO_2_N film photoanode (a) and particle-assembled SrTaO_2_N photoanode (b) recorded at 0.6 V vs. RHE; the inset shows the equivalent circuit model.

Electrochemical impedance spectroscopy measurements were performed in a three-electrode configuration with frequencies ranging from 0.1 Hz to 100 kHz, in order to further comprehend the charge transport of these two SrTaO_2_N photoanodes. The Nyquist plots of the SrTaO_2_N photoanodes (shown in Figure 6) reveal two semicircles. The impedance spectra have been modelled to an equivalent circuit model consisting of two parallel RC elements connected in series, which is shown in the inset of Figure 6(b). The first RC element represents the bulk processes (*R*_bulk_, *C*_bulk_), and the second represents the surface processes (*R*_ct_, *C*_sc_). *R*_s_ is the electrolyte resistance. The fitted results ([Supplementary-material supplementary-material-1]) show that the bulk charge transfer resistance *R*_bulk_ of the SrTaO_2_N film photoanode is only 2% of that of the particle-assembled SrTaO_2_N photoanode, thus resulting in a better PEC performance.

The simplicity and rapidity of this process are applicable to a wide range of oxynitride films. For example, CaTaO_2_N and BaTaO_2_N films were synthesized at the same condition by using the CaCl_2_/CaCO_3_ flux and BaCl_2_/BaCO_3_ flux, respectively, which are proven by their XRD patterns in [Supplementary-material supplementary-material-1]. As-prepared CaTaO_2_N and BaTaO_2_N films are similar crack-free and large-grained films ([Supplementary-material supplementary-material-1]). The HRTEM images of CaTaO_2_N and BaTaO_2_N films display a lattice spacing of 0.280 nm and 0.292 nm ([Supplementary-material supplementary-material-1]), corresponding to CaTaO_2_N (2 0 0) and BaTaO_2_N (1 1 0), respectively. Moreover, SrNbO_2_N and BaNbO_2_N films were also synthesized at the same condition by using Nb foil and corresponding flux (SrCl_2_/SrCO_3_ flux for the SrNbO_2_N film, BaCl_2_/BaCO_3_ flux for the BaNbO_2_N film) as precursors. The XRD patterns confirm the synthesis of SrNbO_2_N and BaNbO_2_N films; the morphology of SrNbO_2_N and BaNbO_2_N films exhibit similar large-grained films ([Supplementary-material supplementary-material-1]). The PEC performance of these films was also measured as shown in [Supplementary-material supplementary-material-1]. The PEC performance of these oxynitride films may be further optimized by choosing the optimal composition of the corresponding flux reagent to generate higher Ca or Ba partial pressure during the nitridation process.

## 3. Discussion

In this study, a series of perovskite oxynitride films such as ATaO_2_N (A = Ca, Sr, and Ba) and ANbO_2_N (A = Sr, Ba) have been prepared by the reactive inorganic vapor deposition method. As-prepared SrTaO_2_N film photoanode exhibits much better PEC performances than the traditional particle-assembled SrTaO_2_N photoanode. Solar water-splitting photocurrent of 3.0 mA cm^−2^ at 1.23 V vs. RHE over the SrTaO_2_N film photoanode is increased by 270%, compared with the highest photocurrent of the particle-assembled SrTaO_2_N photoanode in the literature. Also, the photocurrent remains 86% of its initial activity after AM 1.5G illumination for 5 h, suggesting much better photochemical stability of the SrTaO_2_N film photoanode than the particle-assembled SrTaO_2_N photoanode. The improvement in the PEC performances may be ascribed to less bulk resistance of the directly prepared SrTaO_2_N films. The concept of reactive inorganic vapor deposition growth may cast light on the fabrication of oxide, oxyhalide, and oxysulfide films, as well as other oxynitride films.

## 4. Materials and Methods

### 4.1. Preparation of SrTaO_2_N, BaTaO_2_N, and CaTaO_2_N Films

Ta foils (10 mm × 15 mm × 0.2 mm, ZhongNuo Advanced Material Co., 99.95%) were cleaned in ethanol and acetone. SrTaO_2_N films were prepared on a Ta foil substrate by a reactive inorganic vapor deposition method under a flow of NH_3_. Briefly, 0.2 g of SrCl_2_/SrCO_3_ eutectic salt, which consisted of 26 mol% SrCO_3_ (Sinopharm Chemical Reagent Co., 99.5%) and 74 mol% SrCl_2_·6H_2_O (Sinopharm Chemical Reagent Co., 99.5%), was placed in an alumina combustion boat. Ta foils were put on the top of the alumina combustion boat in a sealed quartz tube furnace. The Ta foils were then annealed at 950°C for 2 hours under a flow of ammonia gas (flow rate: 250 sccm) at a rate of 10°C/min. The samples were then cooled to room temperature with the flow of ammonia gas. After the ammonolysis process, the obtained SrTaO_2_N photoanodes were washed with sufficient deionized water and dried overnight in air before testing. BaTaO_2_N and CaTaO_2_N films were synthesized by using the corresponding flux agents of BaCl_2_ (74%)/BaCO_3_ (26%) and CaCl_2_ (74%)/CaCO_3_ (26%), respectively. SrNbO_2_N and BaNbO_2_N films were synthesized at the same condition by using Nb foil as a precursor and corresponding flux of SrCl_2_ (74%)/SrCO_3_ (26%) and BaCl_2_ (74%)/BaCO_3_ (26%), respectively.

### 4.2. Photoassisted Electrodeposition of Co/CoOOH Catalysts

A two-step electrodeposition method [36] was introduced to deposit a Co/CoOOH catalyst layer from a solution of 10 mM CoSO_4_ and 40 mM potassium acetate. First, 4 mC cm^−2^ of cobalt was electrodeposited at -0.8 V vs. SCE. Then, the 4 mC cm^−2^ of the CoOOH layer was electrodeposited in chronopotentiometry mode at 0.05 mA for 80 s under illumination of a xenon lamp (Ushio, Optical Module X500).

### 4.3. Structural Characterization

The crystal structures of all the samples were measured by X-ray diffraction (XRD, Rigaku Ultima III) with Cu K*α* radiation (*λ* = 1.54056 Å) at 40 kV and 40 mA. The optical absorption spectra of the SrTaO_2_N photoanodes were measured with a UV-visible (UV-vis) spectrophotometer (Shimadzu, UV-vis 2550). The optical band edge of the samples was determined by Tauc plots. The morphology of the films was observed by field-emission scanning electron microscopy (FE-SEM, Nova NanoSEM 230, FEI). TEM and HRTEM analysis was performed on a high-resolution transmission electron microscope (JEM-200CX). TG-DTA coupling mass spectroscopy was carried out on TG-MS equipment (NETZSCH, STA 449 F3-QMS 403 C Aëolos, Germany) in the flow of mixed N_2_/NH_3_ (4 mol%).

### 4.4. PEC Characterization

The PEC activity of SrTaO_2_N photoanodes was measured in a three-electrode configuration cell with a potentiostat (CHI633C) using 1 M NaOH (pH = 13.6) as the electrolyte, the SrTaO_2_N photoanodes as a working electrode, Hg/Hg_2_Cl_2_ in saturated KCl as the reference electrode, and Pt foil as the counter electrode. The CoOOH catalyst was activated by scanning from the open-circuit potential to 2.5 V vs. RHE in 1 M NaOH before the PEC measurements [36]. Measured potentials vs. Hg/Hg_2_Cl_2_ reference electrode were calibrated to reversible hydrogen electrode (RHE). AM 1.5G simulated sunlight (100 mW cm^−2^) was obtained from a Newport Sol3A Class AAA simulator, and the light intensity was calibrated at 100 mW cm^−2^ by a standard reference Newport 91150 silicon cell before testing. The backsides of the SrTaO_2_N films were covered with AB glue and dried overnight before the PEC testing. The wavelength dependence of the incident photon-to-current efficiency (IPCE) was measured under monochromatic light irradiation from a xenon lamp (Ushio, Optical Module X500) equipped with 330-600 nm band pass filters. The light intensity of each wavelength was obtained with a photometer (Newport, 840-C). The applied bias photon-to-current efficiency (ABPE) was calculated from the *J*-*V* curve of the photoanode under AM 1.5G simulated sunlight.

### 4.5. Electrochemical Impedance Spectroscopy Measurements

Electrochemical impedance spectroscopy measurements were performed using an electrochemical analyzer (Solartron 1260+1287) with a frequency range of 0.1 Hz-100 kHz. All the measurements were obtained at room temperature at an applied potential of 0.6 V vs. RHE under dark conditions. The EIS spectral data were fitted to the electrical analogue using ZView software.

## Supplementary Materials

Supplementary Materials Figure S1: phase diagram of the system SrCl_2_-SrCO_3_. Figure S2: top-down SEM image of the SrTaO_2_N film. Figure S3: SAED pattern of the SrTaO_2_N film. Figure S4: *I*-*V* characteristics of the SrTaO_2_N film. Figure S5: XRD patterns of control experiments. Figure S6: XPS spectra from 220 eV to 180 eV. Figure S7: XPS spectra of C1s. Figure S8: TEM and HRTEM images of the peeled SrTaO2N particles. Figure S9: photocurrents of the SrTaO_2_N film prepared with different compositions of the SrCl_2_/SrCO_3_ flux reagent. Figure S10: SEM image of the CoOOH catalyst layer. Figure S11: UV-vis diffuse reflectance spectra and Tauc plot of the SrTaO_2_N film. Figure S12: derived photocurrent of the SrTaO_2_N film. Figure S13: ABPE of SrTaO_2_N film photoanode and particle-assembled SrTaO_2_N photoanode. Figure S14: Mott-Schottky plots of the SrTaO_2_N film. Figure S15: XRD patterns of CaTaO_2_N and BaTaO_2_N films. Figure S16: SEM images of CaTaO_2_N and BaTaO_2_N films. Figure S17: HRTEM images of CaTaO_2_N and BaTaO_2_N films. Figure S18: XRD patterns and SEM images of SrNbO_2_N and BaNbO_2_N films. Figure S19: *J*-*V* curves of the CaTaO_2_N film, BaTaO_2_N film, SrNbO_2_N film, and BaNbO_2_N film. Table S1: the fitted values of *R*_bulk_. Table S2: Water oxidation onset potentials and solar photocurrents of SrTaO_2_N photoanodes.
