# Supplementary material for: Reactive Inorganic Vapor Deposition of Perovskite Oxynitride Films for Solar Energy Conversion
Source: Research (Wash D C). 2019 Nov 11;2019:9282674. doi: 10.34133/2019/9282674 (PMC6946269; doi:10.34133/2019/9282674)
Supplement: Supplementary Materials — Figure S1: phase diagram of the system SrCl2-SrCO3. Figure S2: top-down SEM image of the SrTaO2N film. Figure S3: SAED pattern of the SrTaO2N film. Figure S4: I-V characteristics of the SrTaO2N film. Figure S5: XRD patterns of control experiments. Figure S6: XPS spectra from 220 eV to 180 eV. Figure S7: XPS spectra of C1s. Figure S8: TEM and HRTEM images of the peeled SrTaO2N particles. Figure S9: photocurrents of the SrTaO2N film prepared with different compositions of the SrCl2/SrCO3 flux reagent. Figure S10: SEM image of the CoOOH catalyst layer. Figure S11: UV-vis diffuse reflectance spectra and Tauc plot of the SrTaO2N film. Figure S12: derived photocurrent of the SrTaO2N film. Figure S13: ABPE of SrTaO2N film photoanode and particle-assembled SrTaO2N photoanode. Figure S14: Mott-Schottky plots of the SrTaO2N film. Figure S15: XRD patterns of CaTaO2N and BaTaO2N films. Figure S16: SEM images of CaTaO2N and BaTaO2N films. Figure S17: HRTEM images of CaTaO2N and BaTaO2N films. Figure S18: XRD patterns and SEM images of SrNbO2N and BaNbO2N films. Figure S19: J-V curves of the CaTaO2N film, BaTaO2N film, SrNbO2N film, and BaNbO2N film. Table S1: the fitted values of Rbulk. Table S2: Water oxidation onset potentials and solar photocurrents of SrTaO2N photoanodes. [file 9282674.f1.docx]

**Supplementary Materials for**

**Reactive inorganic vapor deposition of perovskite oxynitride films for solar-energy conversion**

Tao Fang^1^, Huiting Huang^1,2^, Jianyong Feng^1,2^, Yingfei Hu^1,2^, Qinfeng Qian^1,2^, Shicheng Yan^1,2,3^, Zhentao Yu^1,2,3^, Zhaosheng Li^1,2,3,*^, and Zhigang Zou^1,2,3^

^1^ Collaborative Innovation Center of Advanced Microstructures, National Laboratory of Solid State Microstructures, Department of Physics, Nanjing University, 22 Hankou Road, Nanjing 210093, People's Republic of China.

^2^ College of Engineering and Applied Sciences, Nanjing University, 22 Hankou Road, Nanjing 210093, People's Republic of China.

^3^ Jiangsu Key Laboratory for Nano Technology, Nanjing University, 22 Hankou Road, Nanjing 210093, People's Republic of China.

^*^ Correspondence should be addressed to Zhaosheng Li: zsli@nju.edu.cn

**Contents:**

Figure S1 - S19

Table S1 - S2

**Supplementary Figures**


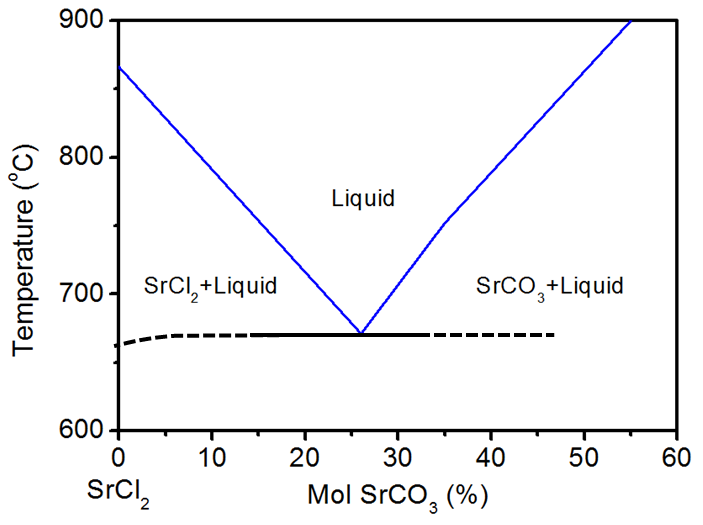


**Figure S1. Phase diagram of the system SrCl_2_−SrCO_3_.** Phase diagram of the system SrCl_2_−SrCO_3_.^[1]^


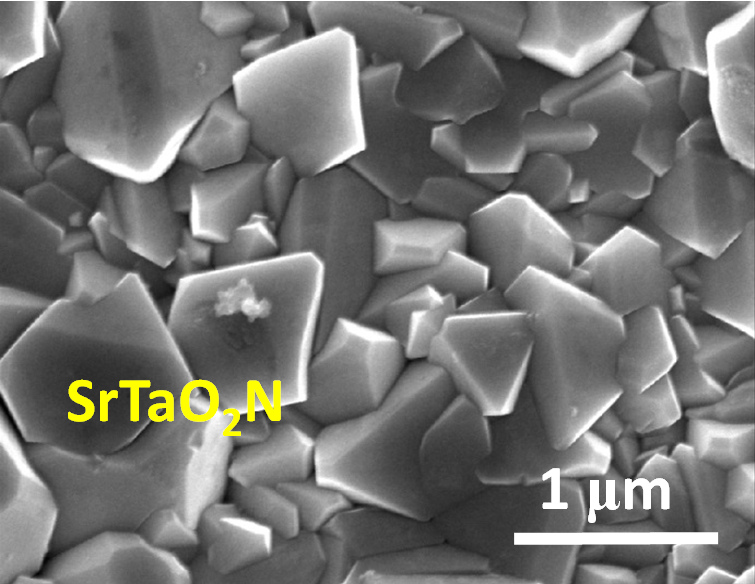


**Figure S2. Top-down SEM image of SrTaO_2_N film.** Top-down SEM image of SrTaO_2_N film.


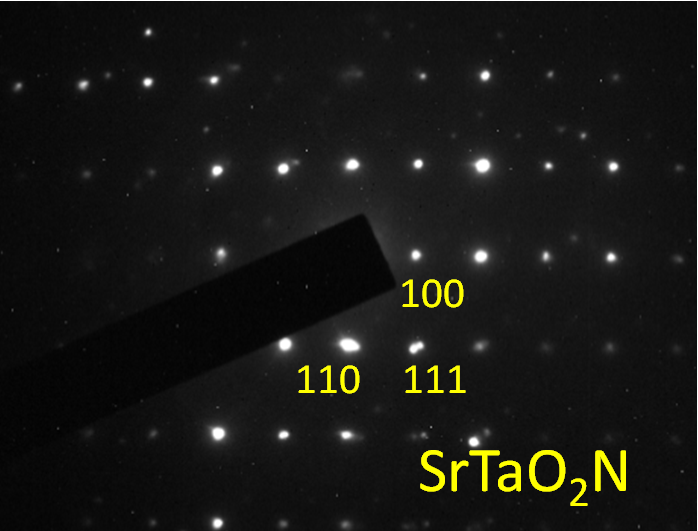


**Figure S3. SAED pattern of the SrTaO_2_N film.** Selected area electron diffraction pattern (SAED) of the directly prepared SrTaO_2_N film.


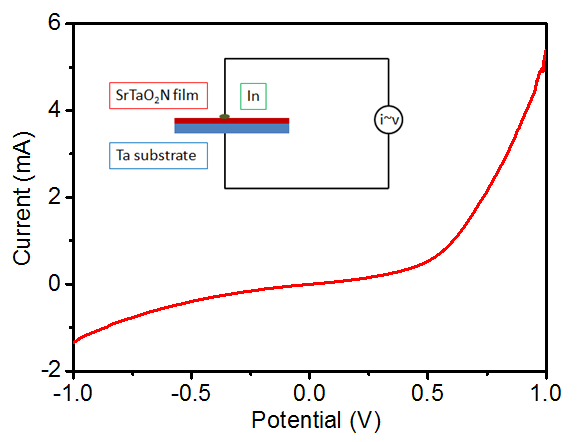


**Figure S4. I-V characteristics of the SrTaO_2_N film.** I-V characteristics of the SrTaO_2_N film. Inset: schematic illustration for the I-V measurement. Condition: room temperature; in dark.


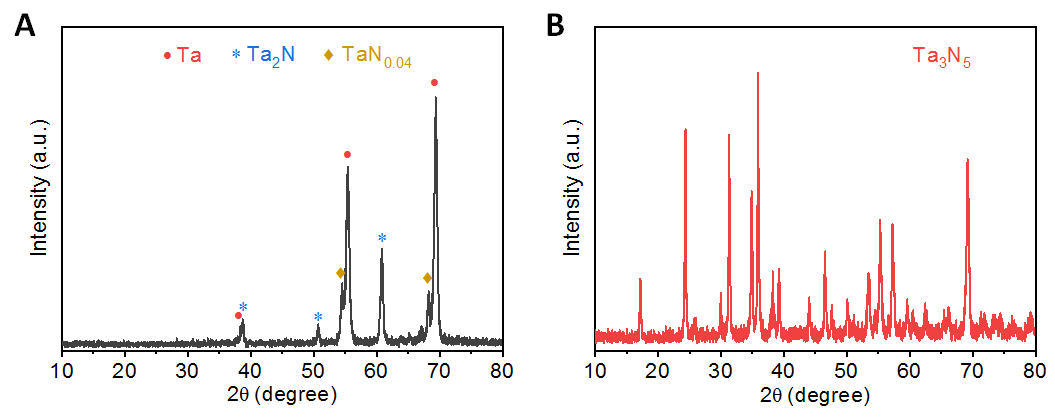


**Figure S5. XRD patterns of control experiments.** XRD patterns of samples prepared in the same condition with only SrCl_2_ as flux agent (A) and only SrCO_3_ as flux agent (B).


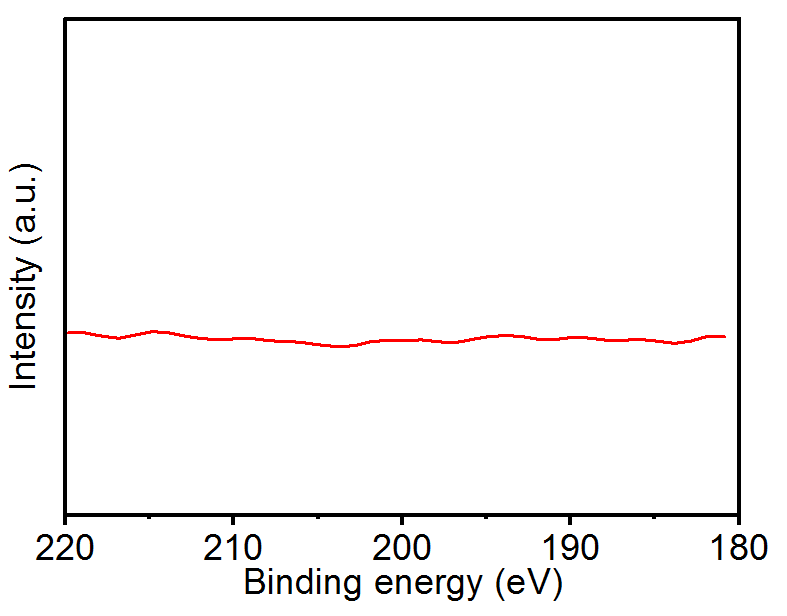


**Figure S6.** **XPS** **spectra from 220 eV to 180 eV.**


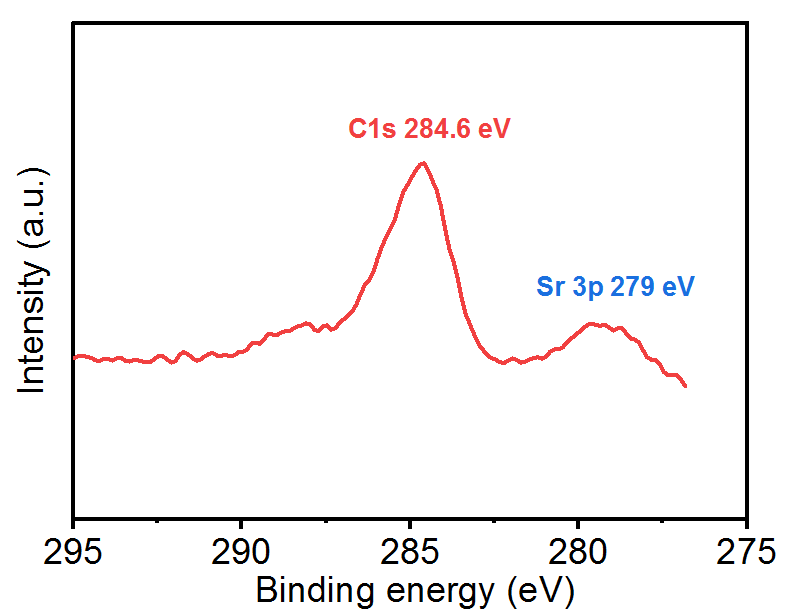


**Figure S7.** **XPS spectra of C 1s.** XPS spectra of C 1s for the SrTaO_2_N film. The atomic concentration of C is about 33.6 %.


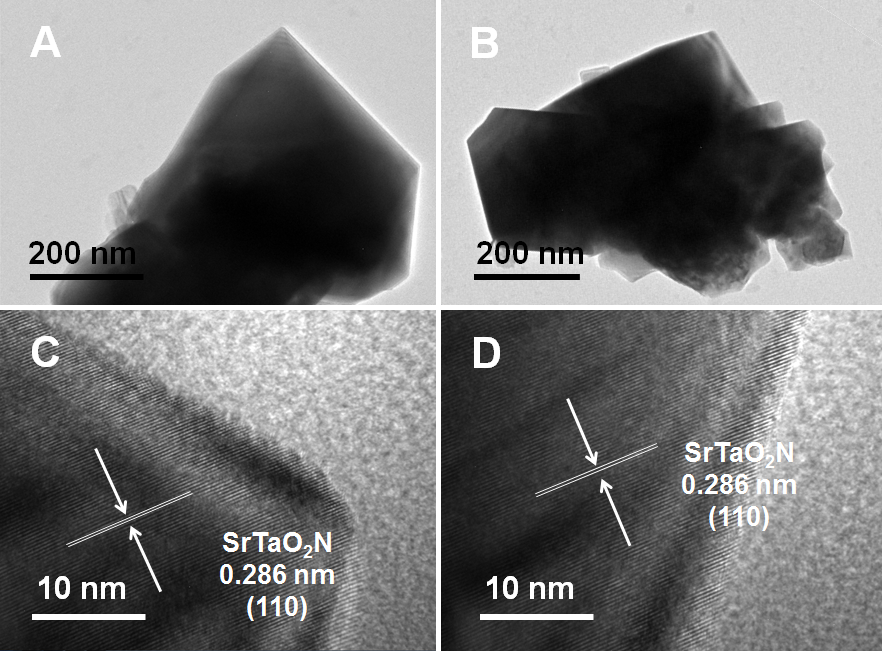


**Figure S8.** **TEM and HRTEM images of the peeled SrTaO_2_N particles.** TEM images (A and B) and HRTEM images (C and D) of the peeled SrTaO_2_N particles.


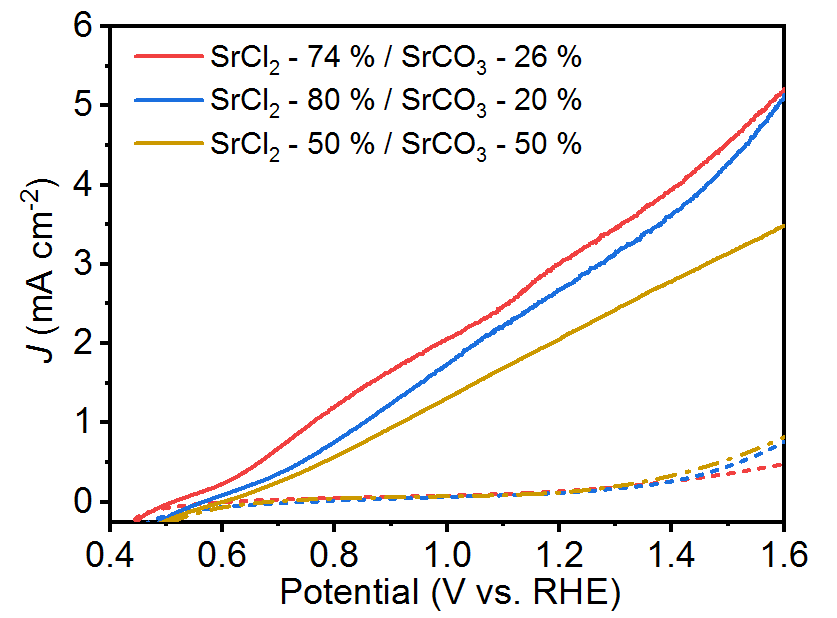


**Figure S9.** **Photocurrents of SrTaO_2_N film prepared with different composition of SrCl_2_/SrCO_3_ flux reagent.** Photocurrents of SrTaO_2_N film prepared with different composition of SrCl_2_/SrCO_3_ flux reagent in 1 M NaOH (pH = 13.6) electrolyte under AM 1.5G (100 mW cm^-2^) simulated sunlight and a scan rate of 30 mV s^-1^.


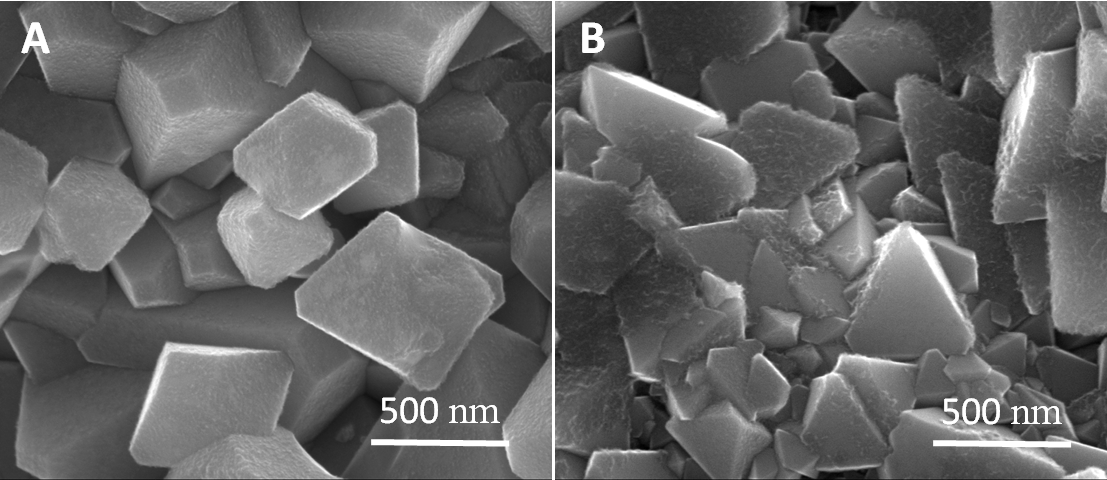


**Figure S10.** **SEM image of the CoOOH catalyst layer.** SEM image of the uniform CoOOH catalyst layer grown on the SrTaO_2_N film photoanode before (a) and after (b) the PEC stability testing. The photochemical stability testing was measured in 1 M NaOH (pH = 13.6) at 1.23 V vs. RHE under AM 1.5 G (100 mW cm^-2^) illumination of 4 hours.


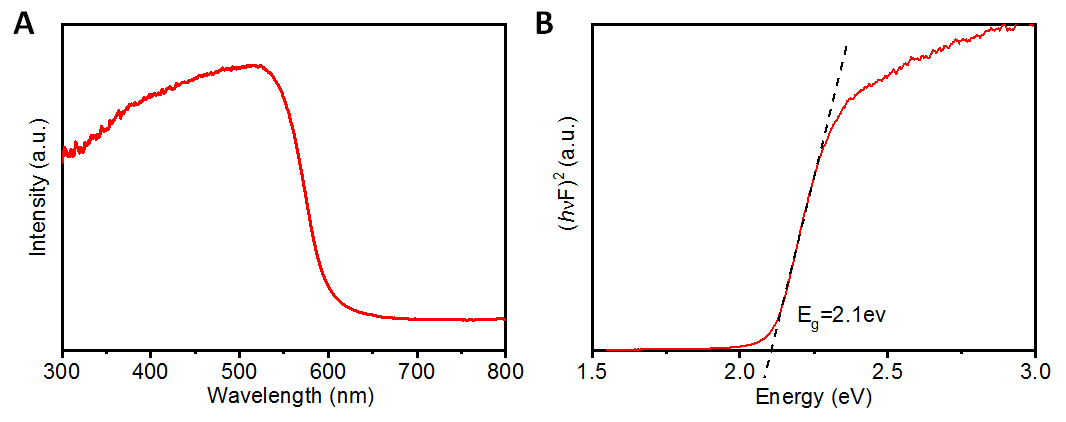


**Figure S11. UV-vis diffuse reflectance spectra and Tauc Plot of the SrTaO_2_N film.** (a) UV-vis diffuse reflectance spectra and (b) Tauc Plot of the SrTaO_2_N film.


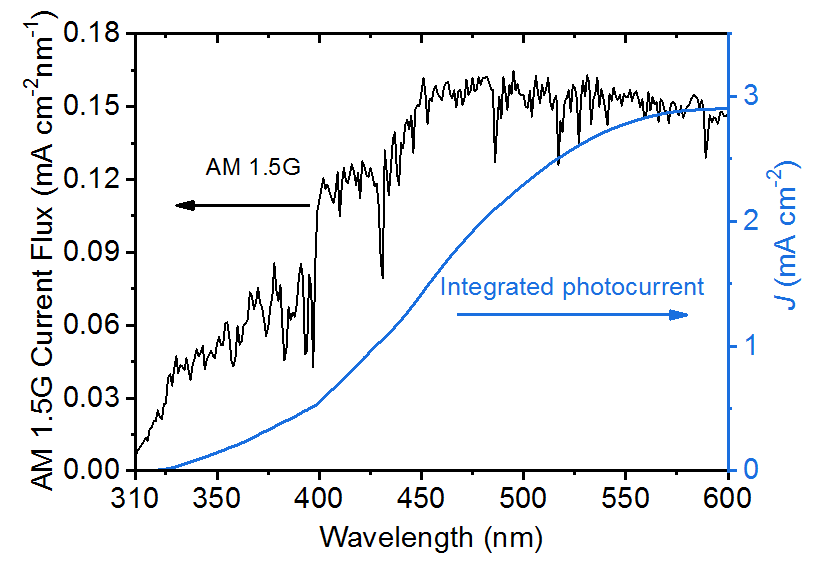


**Figure S12. derived photocurrent of the SrTaO_2_N film.** Standard AM 1.5G solar current flux (black curve) and derived photocurrent (blue curve) of the SrTaO_2_N film at 1.23 V vs. RHE calculated from integrating IPCE spectra over the AM 1.5G standard solar spectral distribution (100 mW cm^-2^).

**Figure S13. ABPE of SrTaO_2_N** **film photoanode and particle-assembled SrTaO_2_N photoanode.** The applied bias photon-to-current efficiency (ABPE) of SrTaO_2_N film photoanode and particle-assembled SrTaO_2_N photoanode^[2]^ under AM 1.5G simulated sunlight (100 mW cm^-2^) in 1 M NaOH (pH = 13.6) aqueous solution.

**Figure S14. Mott-Schottky plots of the SrTaO_2_N film.** Mott-Schottky plots of the SrTaO_2_N films at frequencies of 200, 500, 750 and 1000 Hz.


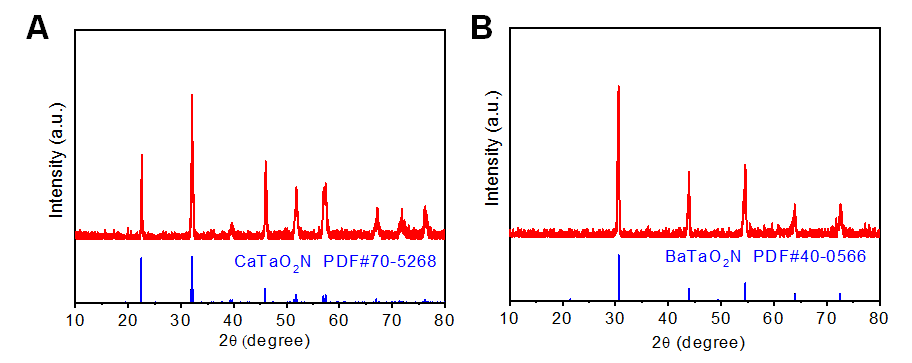


**Figure S15. XRD patterns of CaTaO_2_N and** **BaTaO_2_N films.** (B). XRD patterns of CaTaO_2_N film (A) and BaTaO_2_N film (B).


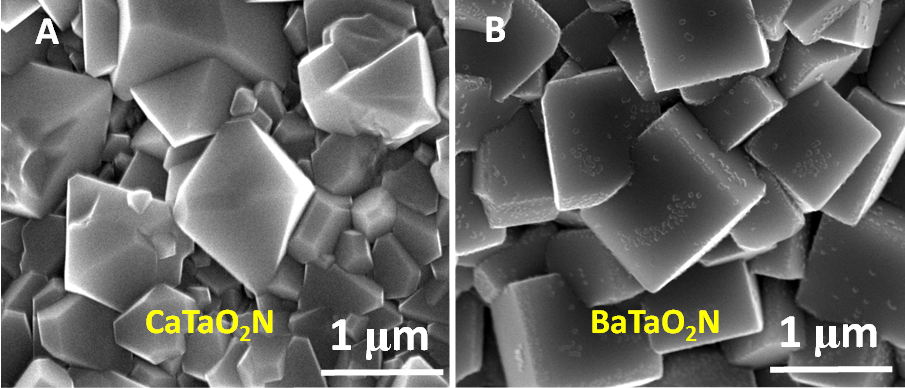


**Figure S16.** **SEM images of CaTaO_2_N and** **BaTaO_2_N films.** Top-down SEM images of CaTaO_2_N film (A) and BaTaO_2_N film (B).


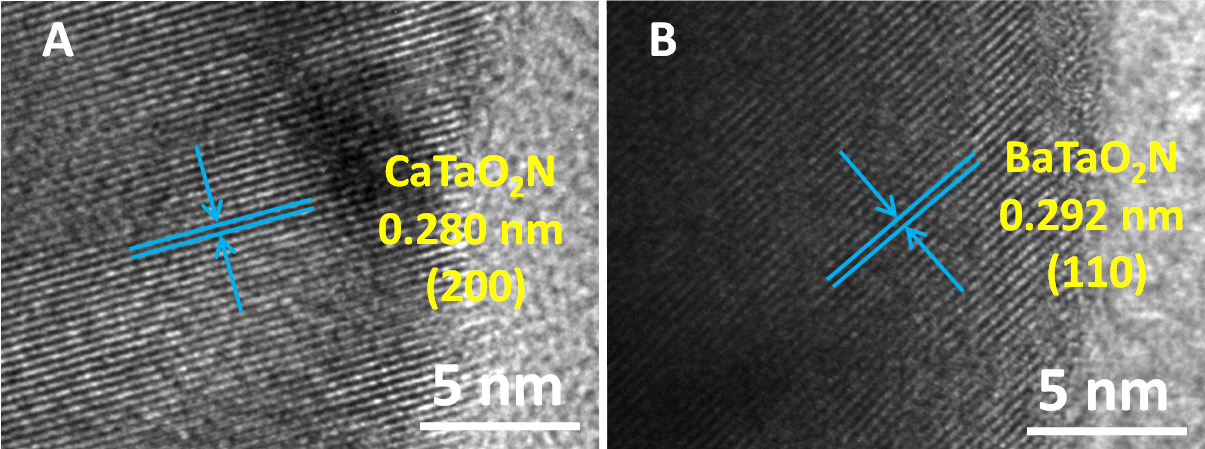


**Figure S17.** **HRTEM images of CaTaO_2_N and** **BaTaO_2_N films.** HRTEM images of CaTaO_2_N film (A) and BaTaO_2_N film (B).


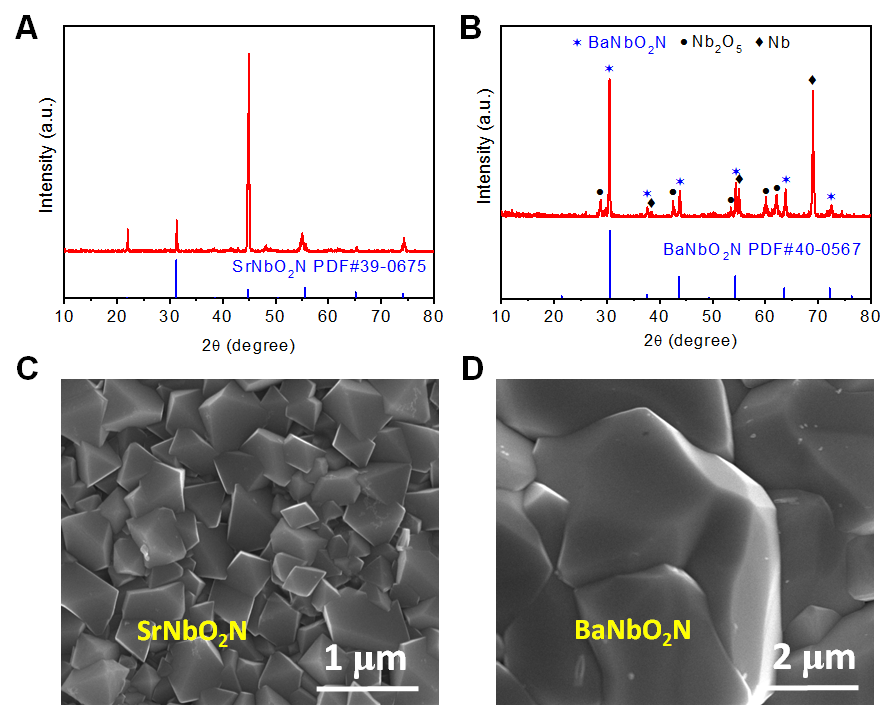


**Figure S18. XRD patterns and SEM images of SrNbO_2_N and** **BaNbO_2_N films.**  XRD patterns of SrNbO_2_N film (A) and BaNbO_2_N film (B). Top-down SEM images of SrNbO_2_N film (A) and BaNbO_2_N film (B).


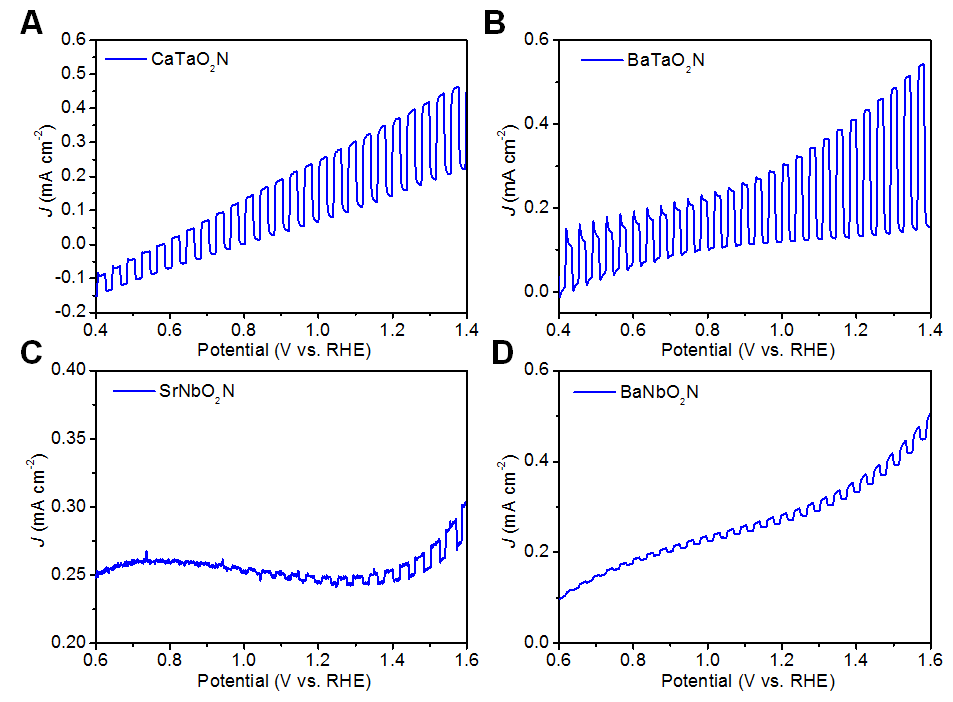


**Figure S19. J-V curves of CaTaO_2_N film, BaTaO_2_N film, SrNbO_2_N film and BaNbO_2_N film.** J-V curves of CaTaO_2_N film (A), BaTaO_2_N film (B), SrNbO_2_N film (C) and BaNbO_2_N film (D) in 1 M NaOH (pH = 13.6) electrolyte under AM 1.5G (100 mW cm^-2^) simulated sunlight and a scan rate of 30 mV s^-1^.

**Supplementary Tables**

**Table S1. The fitted values of R_bulk_.** The fitted values of R_bulk_ for the particle-assembled SrTaO_2_N photoanode and SrTaO_2_N film photoanode at 0.6 V vs. RHE.

|  | particle-assembled SrTaO_2_N | | RVD SrTaO_2_N |
| --- | --- | --- | --- |
| R_bulk_ /Ω | | 11178 | 237 |

**Table S2. Water-oxidation onset potentials and solar photocurrents of SrTaO_2_N photoanodes.**

|  | Onset Potential (V vs. RHE) | Photocurrent at 1.23 V vs. RHE (mA/cm^2^) |
| --- | --- | --- |
| RVD SrTaO_2_N photoanode  (this study) | 0.55 | 3.0 |
| particle-assembled SrTaO_2_N photoanode  (Ref. 2) | 0.6 **^[^** ^2^**^]^** | 1.1 **^[^** ^2^**^]^** |
| SrTaO_2_N nanowire photoanode  (Ref. 3) | ~ 1.1 **^[^**^3^**^]^** | ~ 0.05 **^[^** ^3^**^]^** |

REFERENCES

[1] A. M. Martre, and P. Pouillen, " Contribution à la détermination du diagramme binaire SrCl_2_-SrCO_3_," *Comptes Rendus de l'Académie des Sciences*, vol. 263, pp. 1477-1480, 1966.

[2] Y. Zhong, Z. Li, X. Zhao, T. Fang, H. Huang, Q. Qian, X. Chang, P. Wang, S. Yan, Z. Yu, and Z. Zou, "Enhanced water-splitting performance of perovskite SrTaO_2_N photoanode film through ameliorating interparticle charge transport," *Advanced Functional Materials*, vol. 26, no. 39, pp. 7156-7163, 2016.

[3] M. Davi, F. Schrader, T. Scholz, Z. Ma, A. Rokicinska, R. Dronskowski, P. Kustrowski, and A. Slabon, "SrTaO_2_N nanowire photoanode modified with a ferrihydrite hole-storage layer for photoelectrochemical water oxidation," *ACS Applied Nano Materials*, vol. 1, no. 2, pp. 869-876, 2018.
